# Comparative Physiological and Biochemical Responses of Lettuce (*Lactuca sativa* L. var. *romana*) to Foliar Methyl Jasmonate, Nano-Kaolin, and Melatonin Under a Progressive High-Temperature Regime

**DOI:** 10.3390/plants15193013

**Published:** 2026-09-30

**Authors:** Erhan Akalp

**Affiliations:** Department of Horticulture, Faculty of Agriculture, Dicle University, 21280 Diyarbakır, Turkey; erhan.akalp@dicle.edu.tr; Tel.: +90-534-798-2749

**Keywords:** high temperature stress, *Lactuca sativa*, melatonin, methyl jasmonate, nano-kaolin

## Abstract

High temperature is an important environmental constraint affecting lettuce growth and physiological performance. This study comparatively evaluated the responses of lettuce (*Lactuca sativa* L. var. *romana*) to individually applied foliar methyl jasmonate (MeJA; 0.5 and 1.0 mM), nano-kaolin (0.5 and 1.0%), and melatonin (0.1 mM) under a common progressive high-temperature regime. Plants were grown in two contrasting growing media, soil and peat–perlite, and were exposed to temperatures progressively increased from 20 to 40 °C. Growth characteristics, visual damage, leaf temperature, membrane leakage, relative water content (RWC), leaf color, total soluble solids, pH, DPPH and ABTS radical-scavenging capacity, and total phenolic content were evaluated. Treatment responses differed substantially between growing media. In peat–perlite, nano-kaolin treatments produced the highest fresh biomass and relatively low visual damage, whereas responses in soil were more variable among treatments. RWC was significantly affected by treatment in both growing media, although the response pattern differed between substrates. Membrane leakage did not differ significantly among treatments in either growing medium. Nano-kaolin treatments produced comparatively high DPPH and ABTS radical-scavenging capacity and total phenolic content in both growing media. Overall, the findings demonstrate that the physiological and biochemical responses of lettuce to the tested foliar treatments were strongly influenced by the growing medium under the imposed high-temperature regime.

## 1. Introduction

As a consequence of global climate change, the increasing frequency and intensity of sudden, extreme temperature fluctuations represent a primary abiotic stress factor threatening fresh vegetable production and sustainable horticultural practices worldwide [1]. High-temperature waves induce severe negative impacts on pollen viability, fruit set, yield, and market value. Agronomic practices such as grafting, shade netting, and planting date optimization are frequently used to alleviate these adverse effects. Additionally, exogenously applying organic acids, bio-stimulants, hormones, and signaling molecules has emerged as a practical strategy that enhances thermotolerance at the cellular level by activating antioxidant defense systems and osmotic adjustment [2].

Among cool-season vegetables, lettuce (*Lactuca sativa* L.) holds high economic value in fresh consumption but is highly sensitive to elevated temperatures both morphologically and physiologically [3,4]. Optimal growth for lettuce occurs between 15 °C and 20 °C; rapid temperature spikes trigger premature bolting, tip burn, bitterness, and biomass loss [3,4]. Under such conditions, cellular-level regulation of stress-responsive genes and transcription factors plays a crucial role in sustaining heat resistance [5]. When plants undergo sudden heat shock, excessive accumulation of reactive oxygen species (ROS) occurs in organelles such as chloroplasts and mitochondria [6]. The resulting oxidative stress accelerates lipid peroxidation, leading to malondialdehyde (MDA) buildup, disruption of cell membrane integrity, uncontrolled electrolyte leakage, and a rapid decline in relative water content [7]. To protect against cellular injury, lettuce plants activate a range of antioxidant and osmotic adjustment mechanisms that help maintain cellular integrity under heat stress [8]. However, when stress intensity exceeds the genetic tolerance threshold of the plant, foliar application of exogenous protective agents becomes necessary to support cellular homeostasis [9].

The physical and chemical characteristics of the growing medium may substantially influence plant responses to heat stress [10]. Peat-based substrates generally provide higher water-holding capacity, improved aeration, and more stable root-zone conditions, whereas soil-based media more closely represent commercial cultivation environments. Evaluating treatment performance across contrasting growing media may therefore provide a broader assessment of the consistency of foliar treatment responses under a common high-temperature regime.

In recent years, signaling molecules, plant growth regulators, and nanomaterials that trigger plant defense mechanisms through distinct pathways have been extensively studied. Melatonin, a pleiotropic hormone, functions as both a powerful ROS scavenger and a master regulator that upregulates antioxidant gene expression, preserving chlorophyll stability and thermotolerance [11,12]. Similarly, Methyl Jasmonate (MeJA), a lipid-derived signaling molecule, stimulates secondary metabolite production, regulates stomatal movement to prevent transpirational water loss, and enhances abiotic stress resistance [13,14]. Moreover, MeJA applications reportedly induce phenylalanine ammonia-lyase (PAL) activity, promoting phenolic compound accumulation and antioxidant capacity [15]. On the other hand, kaolin nanoparticles (Nano-Kaolin) serve as a physio-mechanical protective film that reflects excessive light and thermal radiation, thereby lowering leaf temperature and preventing overheating of the photosynthetic apparatus [16].

Although the individual effects of melatonin [12], methyl jasmonate [13,15], and kaolin [16] against abiotic stresses have been investigated in various plant species, comparative information on the responses of lettuce to individually applied doses of these three distinct foliar treatments under a progressive high-temperature regime remains limited.

Therefore, the main objective of this study was to compare the relative effectiveness of individually applied foliar MeJA, nano-kaolin, and melatonin on the physiological and biochemical responses of lettuce plants exposed to a common progressive high-temperature regime. A further objective was to determine whether the responses to these foliar treatments differed between two contrasting growing media, soil and peat–perlite, thereby evaluating the consistency of treatment performance across different root-zone environments. Plant growth parameters, membrane integrity, relative water content, visual damage, DPPH and ABTS radical-scavenging capacity, and total phenolic content were evaluated. We hypothesized that the three foliar treatments would produce distinct physiological and biochemical response patterns under the common progressive high-temperature regime and that the magnitude of these responses would differ between the two growing media.

## 2. Results

### 2.1. Morphological and Growth Parameters

Under the imposed high-temperature regime, plant growth responses differed significantly among foliar treatments, particularly in the peat–perlite substrate. In the peat–perlite substrate, fresh plant weight was 1.13 g in the control treatment, whereas significantly higher values were recorded for Melatonin (9.36 g), Kaolin 0.5% (11.15 g), Kaolin 1.0% (11.61 g), and MeJA 1.0 mM (9.86 g) under the common progressive high-temperature regime (*p* < 0.0001) (Table 1). Plant height in the peat control was 10.23 cm, reaching 16.79 cm with Kaolin 1.0% and 16.36 cm with Kaolin 0.5% (*p* = 0.0001). Plant dry weight was also significantly affected by foliar treatment in the peat–perlite substrate (*p* = 0.0255). The highest mean values were recorded in the peat control (1.69 g) and Kaolin 0.5% (1.60 g), whereas the lowest mean value was observed in the Melatonin treatment (0.79 g). MeJA 0.5 mM showed an intermediate response (1.22 g) (Table 1).

In the soil substrate, fresh plant weight reached its maximum value under Melatonin treatment (18.14 g) compared to the soil control (8.69 g) (Table 2). Regarding root parameters in peat substrate, root length increased from 4.74 cm in the control to 8.21 cm in MeJA 1.0 mM (*p* = 0.0009), while root dry weight increased significantly in MeJA 1.0 mM (0.55 g) and Melatonin (0.52 g) compared to the control (0.12 g) (*p* < 0.0001). In the soil substrate, root weight (*p* = 0.1599), root length (*p* = 0.6562), and root diameter (*p* = 0.9214) did not differ significantly among foliar treatments.

### 2.2. Physiological Responses, Visual Damage, and Membrane Integrity

In the peat–perlite substrate, leaf surface temperature ranged from 18.91 °C in the melatonin treatment to 23.03 °C in the control; however, differences among treatments were not statistically significant (*p* = 0.2516) (Table 3). Relative Water Content (RWC %) in peat substrate was 66.70% in the control, remaining higher in MeJA 1.0 mM (72.40%) and MeJA 0.5 mM (68.24%) (*p* = 0.0111). Visual scale damage (0–5 scale) reached 2.71 in the peat control, whereas damage remained minimal in Melatonin (0.00), Kaolin 1.0% (0.00), Kaolin 0.5% (0.43), MeJA 1.0 mM (0.57), and MeJA 0.5 mM (0.71) (*p* < 0.0001).

In the soil substrate, membrane leakage did not differ significantly among treatments (*p* = 0.0931), although numerical differences were observed among treatment means. RWC differed significantly among treatments (*p* = 0.0014), reaching 73.07% in nano-kaolin 1.0%, 72.98% in MeJA 1.0 mM, and 69.32% in MeJA 0.5 mM, compared with 45.71% in the soil control. Leaf b* values differed significantly among treatments (*p* = 0.0098), with the lowest mean value recorded in nano-kaolin 1.0% (16.53), while the soil control, MeJA 0.5 mM, and MeJA 1.0 mM formed the highest statistical group (Table 4).

### 2.3. Radical-Scavenging Capacity and Total Phenolic Content

In the peat substrate, DPPH radical-scavenging capacity was 35.26% in the control, increasing to 47.24% in Kaolin 1.0% and 42.74% in Kaolin 0.5% (*p* < 0.001) (Table 5). Kaolin 1.0% (98.63%) and Kaolin 0.5% (97.44%) also exhibited the highest ABTS radical-scavenging capacity (*p* < 0.001). Total phenolic content reached its maximum under Kaolin 0.5% (0.2980 µg GAE/µL) and Kaolin 1.0% (0.2947 µg GAE/µL) (*p* < 0.001).

Similar trends were observed in the soil substrate (Table 6). DPPH radical-scavenging capacity increased from 31.45% in the control to 45.10% in Kaolin 1.0% (*p* < 0.001). ABTS radical-scavenging capacity was highest in Kaolin 1.0% (96.80%) and Kaolin 0.5% (93.60%) (*p* < 0.001). Total phenolic content reached 0.2712 µg GAE/µL in Kaolin 1.0% and 0.2650 µg GAE/µL in Kaolin 0.5% compared with 0.1985 µg GAE/µL in the soil control (*p* < 0.001).

Overall, the treatment responses were parameter- and growing-medium-dependent. Nano-kaolin was associated with comparatively high fresh biomass in peat–perlite and high DPPH and ABTS radical-scavenging capacity and total phenolic content in both growing media, whereas MeJA 1.0 mM was associated with comparatively high RWC in the soil substrate.

For clearer visualization of the treatment-dependent responses across the two growing media, fresh plant weight, RWC, DPPH radical-scavenging capacity, and total phenolic content are comparatively presented in Figure 1.

## 3. Discussion

Under the imposed progressive high-temperature regime, lettuce plants showed treatment-dependent differences in growth and visual injury, and these responses varied between the two growing media. In the peat–perlite substrate, nano-kaolin treatments produced the highest fresh biomass and relatively low visual damage, whereas responses in soil were less uniform among treatments. Because the present experiment did not include a non-stressed temperature control, these differences should be interpreted as comparative responses among foliar treatments under the same high-temperature regime rather than as a direct quantification of heat-induced growth reduction. Heat stress is known to impair lettuce growth and development through disturbances in cellular homeostasis, water relations, membrane function, and metabolic processes [17,18]. The responses observed here are therefore consistent with the potential stress-modulating roles previously reported for melatonin, methyl jasmonate, and kaolin particle films [11,13,16], including recent evidence of their involvement in plant responses to high-temperature stress [19,20,21], although the underlying mechanisms were not directly measured in the present study.

Relative water content (RWC) is an important indicator of plant water status under environmental stress. In the present study, RWC responses were strongly dependent on both treatment and growing medium. In peat–perlite, MeJA treatments maintained RWC values statistically comparable to the distilled-water control, whereas nano-kaolin 1.0% resulted in the lowest RWC. In contrast, in soil, nano-kaolin 1.0% and both MeJA treatments showed significantly higher RWC than the distilled-water control. These contrasting patterns indicate that the effects of foliar treatments on plant water status were influenced by the root-zone environment rather than representing a uniform treatment response. Previous studies have associated jasmonate-mediated responses with regulation of stomatal behavior and plant water relations [13], while kaolin particle films may modify leaf energy balance and transpiration by increasing surface reflectance [16]. However, because stomatal conductance and transpiration were not directly measured in this study, these mechanisms should be regarded as possible explanations for the observed RWC responses rather than as directly demonstrated effects.

The biochemical responses also differed among foliar treatments. Nano-kaolin treatments showed particularly high DPPH and ABTS radical-scavenging capacity and total phenolic content in both growing media. Kaolin particle films are primarily considered to act physically by increasing leaf-surface reflectance and modifying the radiation and energy balance of leaves [16]. Such physical effects may indirectly influence cellular metabolism under high-temperature conditions; however, leaf temperature did not differ significantly among treatments in the present study. Therefore, the higher radical-scavenging capacity and phenolic content observed following nano-kaolin application cannot be attributed directly to reduced leaf temperature and may involve additional physiological or metabolic responses that were not measured in this experiment.

Methyl jasmonate is a signaling molecule involved in plant responses to abiotic stress and has been associated with regulation of secondary metabolism and phenolic biosynthesis [13,14,22]. In the present study, however, the biochemical response to MeJA was less pronounced than that observed with nano-kaolin. Thus, the DPPH, ABTS, and total phenolic results should be interpreted as treatment-dependent changes in the in vitro radical-scavenging properties and phenolic content of lettuce extracts rather than as direct evidence of activation of specific antioxidant enzymes or metabolic pathways. Measurements of phenylalanine ammonia-lyase activity, antioxidant enzymes, reactive oxygen species, and lipid peroxidation would be required to confirm these proposed mechanisms.

The contrasting responses observed between peat–perlite and soil indicate that the growing medium influenced the effectiveness of the foliar treatments. Growing media differ substantially in their physical and hydraulic properties, particularly in water-holding capacity, aeration, porosity, and nutrient availability, which can modify root-zone conditions and consequently influence above-ground plant responses [10]. Peat-based substrates generally provide high water-holding capacity, whereas the incorporation of perlite can improve drainage and aeration, thereby creating a root-zone environment that differs substantially from mineral soil [10]. In the present study, the stronger biomass response to nano-kaolin in peat–perlite and the comparatively different responses observed in soil may therefore be associated with differences in root-zone water and air relations. However, because detailed physicochemical and hydraulic properties of the two growing media were not determined, the specific mechanisms responsible for these substrate-dependent responses cannot be established from the present experiment. Further studies combining foliar treatments with measurements of substrate water-holding capacity, porosity, electrical conductivity, nutrient availability, and root-zone temperature would help clarify these interactions.

Although numerical reductions in membrane leakage were observed following some treatments, particularly in the soil substrate, these differences were not statistically significant in either growing medium. Therefore, the present results do not provide evidence of a significant treatment-induced improvement in membrane stability. In contrast, significant treatment effects were detected for DPPH and ABTS radical-scavenging capacity and total phenolic content. These assays characterize the in vitro radical-scavenging properties and phenolic content of plant extracts and should not be interpreted as direct measurements of the in vivo antioxidant enzyme system. The relationship between phenolic accumulation and radical-scavenging activity has previously been demonstrated in lettuce, supporting the interpretation that variation in phenolic composition may contribute to differences in extract-based antioxidant activity [23]. However, because reactive oxygen species, lipid peroxidation, osmolytes, and antioxidant enzymes such as SOD, CAT, APX, and POD were not measured in the present study, the biochemical mechanisms underlying the treatment responses cannot be conclusively established. Future studies incorporating these measurements would provide a more comprehensive assessment of oxidative stress and antioxidant defense under high-temperature conditions. The higher fresh weight observed in the soil control relative to some treated groups may reflect treatment-specific responses within the soil root-zone environment, rather than a uniform beneficial effect of the foliar applications. Differences in substrate-dependent water availability, aeration, and root–shoot interactions may have contributed to these contrasting responses.

Among the tested treatments, nano-kaolin consistently produced the highest DPPH and ABTS radical-scavenging capacity and total phenolic content in both growing media. This consistent biochemical response suggests that nano-kaolin application was associated with changes in the extractable phenolic pool and radical-scavenging properties of lettuce leaves under the imposed high-temperature regime. Kaolin particle films are known primarily for their physical effects on leaf optical properties and energy balance [16]; however, the present study does not establish a direct causal relationship between these physical effects and the observed biochemical responses. In particular, leaf temperature did not differ significantly among treatments, and photosynthetic performance was not directly measured. Therefore, the higher DPPH, ABTS, and total phenolic values observed in nano-kaolin-treated plants should be interpreted as treatment-associated biochemical responses rather than direct evidence of improved photosynthetic efficiency or reduced oxidative damage.

This interpretation is also consistent with the findings of Wei et al. [24], who reported that phenolic and flavonoid biosynthesis is involved in the response of lettuce to heat stress. Nevertheless, the present study did not investigate gene expression or individual phenolic compounds; therefore, direct involvement of these metabolic pathways cannot be confirmed from the current data.

From a practical perspective, the comparatively favorable responses observed with nano-kaolin and selected MeJA treatments suggest their potential for further evaluation as foliar management approaches in lettuce exposed to high-temperature conditions, particularly under different growing-media environments. An important limitation of the present study is the absence of a non-stressed temperature control maintained under optimal temperature conditions. Therefore, the experimental design does not allow direct quantification of the magnitude of heat-induced injury or the degree of heat-stress alleviation relative to non-stressed plants. Rather, the results demonstrate comparative responses of individually applied foliar treatments within a common progressive high-temperature regime. In addition, oxidative stress markers and antioxidant enzyme activities were not directly determined. Additionally, the distilled-water control did not contain Tween-20 or ethanol, whereas Tween-20 was included in the treatment solutions and ethanol was used as an initial solvent for MeJA and melatonin. Therefore, potential vehicle and surfactant effects cannot be completely excluded. Consequently, the physiological and biochemical mechanisms proposed to explain the treatment responses should be considered literature-supported interpretations rather than mechanisms directly demonstrated by the present experiment. Future studies should include both non-stressed and heat-stressed controls and incorporate measurements of reactive oxygen species, lipid peroxidation, osmolytes, and antioxidant enzymes to more comprehensively determine the mechanisms underlying the responses to MeJA, nano-kaolin, and melatonin. The main contribution of this study is the side-by-side comparison of individually applied methyl jasmonate, nano-kaolin, and melatonin in lettuce under a common progressive high-temperature regime, while also demonstrating that the magnitude and direction of treatment responses can vary between contrasting growing media.

## 4. Materials and Methods

### 4.1. Plant Material and Growth Conditions

This study was conducted in February–March 2026 in controlled plant growth cabinets at the Department of Horticulture, Faculty of Agriculture, Dicle University, Turkey. Homogeneous seedlings of Yedikule lettuce (*Lactuca sativa* L. var. *romana*) obtained from a commercial producer in Antalya were used as plant material. Seedlings were planted into 5 L pots containing either soil or a 2:1 (*v*/*v*) sterile peat: perlite mixture.

Two growing media were intentionally selected to represent contrasting root-zone environments. The peat–perlite substrate represented a highly aerated and water-retentive horticultural medium commonly used in protected cultivation systems, whereas the soil substrate represented conditions more closely associated with practical production environments. This comparison allowed assessment of whether treatment efficacy remained consistent under different substrate conditions.

Environmental conditions were maintained at a 16 h light/8 h dark photoperiod under LED growth lighting, 20 °C ambient temperature, and 60–65% relative humidity. Pots were irrigated regularly and fertigated with balanced Hoagland nutrient solution [25].

### 4.2. Experimental Design

The experiment was arranged in a Completely Randomized Design (CRD) with 3 replications. Each replication comprised 15 pots (one plant per pot), resulting in 45 plants per treatment group for each substrate (45 plants/treatment for soil; 45 plants/treatment for peat:perlite). Pots were rotated randomly every three days to ensure uniform microclimatic exposure.

### 4.3. Exogenous Chemical Treatments and Heat Stress Protocol

Methyl jasmonate (MeJA), melatonin, and nano-kaolin used in the foliar treatments were obtained from Sigma-Aldrich. Methyl jasmonate (MeJA) and melatonin were initially dissolved in ethanol and subsequently diluted with distilled water to obtain the required treatment concentrations. Nano-kaolin suspensions were prepared using distilled water. Tween-20 was added as a surfactant to the MeJA, nano-kaolin, and melatonin treatment solutions. One week after transplanting and 24 h prior to heat exposure, the treatment solutions were applied as foliar sprays until the leaf surfaces were thoroughly wetted. The control plants were sprayed with distilled water only, without Tween-20. The treatment groups were:

- Control (K): Foliar spray with distilled water only.
- Methyl Jasmonate (MeJA): Applied at 0.5 and 1.0 mM. The 0.5 mM concentration was selected based on previous studies reporting physiological and biochemical responses of lettuce to exogenous MeJA [15], while 1.0 mM was included as a higher concentration to evaluate the concentration-dependent response under the imposed high-temperature regime.
- Nano-Kaolin (NK): Applied at 0.5% and 1.0% (*w*/*v*). These two concentrations were selected as two application levels to compare the response of lettuce to nano-kaolin under the imposed high-temperature regime. The use of kaolin was based on its established function as a reflective particle film capable of modifying leaf-surface radiation and energy balance [16].
- Melatonin (M): Applied at 0.1 mM based on the concentration previously used under high-temperature stress conditions [12].

Twenty-four hours after foliar treatment, the temperature was progressively increased according to the following regime: 25 °C for 24 h (Day 1), 30 °C for 24 h (Day 2), 35 °C for 24 h (Day 3), and 40 °C for 24 h (Day 4). Following completion of the progressive high-temperature regime, plants were maintained for a 72 h recovery period prior to sampling.

All treatment groups, including the distilled-water control, were subjected to the same progressive high-temperature regime. No parallel non-stressed group maintained continuously at the initial 20 °C growth temperature was included in the experimental design.

### 4.4. Measurements and Sampling

After the 72 h recovery period, 10 plants were selected from each of the three replicates for each treatment within each growing medium, resulting in a total of 30 sampled plants per treatment per growing medium (10 plants × 3 replicates). The remaining plants were retained as reserve experimental units and were not included in the measurements reported in this study. Relative water content (RWC) was determined using fresh weight (FW), turgid weight (TW), and dry weight (DW) of leaf samples. Fresh weight was recorded immediately after sampling. The samples were then fully hydrated to obtain turgid weight, followed by drying to constant weight for determination of dry weight. RWC was calculated as: RWC (%) = [(FW − DW)/(TW − DW)] × 100. Leaf samples intended for biochemical analyses were initially snap-frozen in liquid nitrogen and stored at −80 °C until further processing. Prior to spectrophotometric analyses, the samples were oven-dried and ground to obtain a homogeneous material for extraction.

Morphological parameters (plant height, plant fresh/dry weight, root length, root diameter, root fresh/dry weight), physiological parameters (leaf surface temperature, leaf color L*, a*, b*, visual scale 0–5, membrane leakage %, RWC %), and basic biochemical parameters (TSS, sap pH) were analyzed at Dicle University. Total soluble solids (TSS) were determined using a PAL-1 digital refractometer (ATAGO, Tokyo, Japan; 0–53 °Brix) and expressed as °Brix. Leaf surface temperature was measured using a non-contact infrared thermometer (UT300S, UNI-T) and expressed as °C. Membrane leakage was determined using the electrical conductivity method. Leaf samples were immersed in distilled water, and the initial electrical conductivity (EC_1_) was measured using a C-600 7-in-1 multiparameter meter (pH/TDS/EC/ORP/salinity/temperature). The samples were subsequently heated in an oven at 100 °C for 25 min to achieve complete electrolyte release. After heat treatment, the final electrical conductivity (EC_2_) was measured. Membrane leakage was calculated as (EC_1_/EC_2_) × 100 and expressed as a percentage. Visual heat-injury symptoms were evaluated using a 0–5 ordinal scale based on the overall severity of leaf wilting, curling, chlorosis, necrosis, and tissue damage. A score of 0 represented plants with no visible injury, whereas a score of 5 represented plants showing the most severe visible injury. Intermediate scores (1–4) represented progressively increasing symptom severity.

Spectrophotometric antioxidant assays were performed at Siirt University Central Research Laboratory. Three independent biological samples were analyzed for each treatment in each growing medium (*n* = 3), and each biological sample was measured in three technical replicates for the spectrophotometric assays. Oven-dried, ground leaf samples were extracted with 75% methanol-0.1% formic acid. DPPH radical-scavenging capacity was determined at 517 nm using Trolox as a standard [26]. ABTS radical-scavenging capacity was determined at 734 nm using the radical cation decolorization assay [27]. Total phenolic content (TPC) was determined using the Folin-Ciocalteu method at 760 nm and expressed as gallic acid equivalents (µg GAE/µL) [28,29].

### 4.5. Statistical Analysis

Statistical analyses were performed separately for each growing medium. Data were subjected to one-way analysis of variance (ANOVA) using JMP software (Version 7.0), with foliar treatment considered as the main factor. When the ANOVA indicated significant treatment effects, mean comparisons were performed using the Least Significant Difference (LSD) test. Statistical significance was evaluated at *p* < 0.05, while *p* < 0.01 was considered highly significant. Data are presented as mean ± standard error (SE).

## 5. Conclusions

This study demonstrated that lettuce responses to individually applied foliar methyl jasmonate, nano-kaolin, and melatonin differed under a common progressive high-temperature regime and were strongly influenced by the growing medium. In peat–perlite, nano-kaolin treatments produced comparatively high fresh biomass and relatively low visual damage, whereas treatment responses in soil were more variable. Relative water content was significantly affected by treatment in both growing media, although the response pattern differed between substrates. In contrast, membrane leakage did not differ significantly among treatments in either growing medium. Nano-kaolin consistently produced the highest DPPH and ABTS radical-scavenging capacity and total phenolic content in both substrates. These findings indicate that treatment performance under elevated-temperature conditions depends on both the foliar treatment and the root-zone environment. However, because a non-stressed temperature control was not included, the present results represent comparative treatment responses within the imposed high-temperature regime and do not quantify the magnitude of heat-stress alleviation relative to plants maintained under optimal temperature conditions. From a practical perspective, the comparatively favorable responses observed with nano-kaolin and selected MeJA treatments support their further evaluation as foliar management approaches for lettuce production under high-temperature conditions. Future studies incorporating non-stressed and heat-stressed controls, oxidative stress markers, antioxidant enzyme activities, and detailed growing-medium characterization are required to verify the mechanisms underlying these responses and their potential practical relevance.

## Figures and Tables

**Figure 1:**
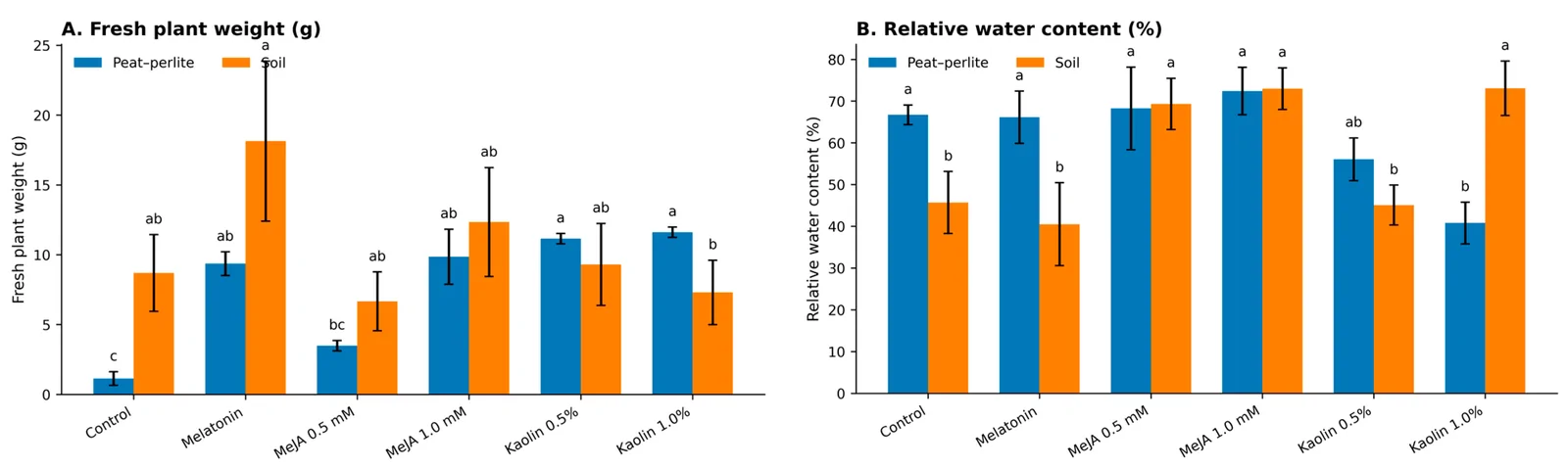

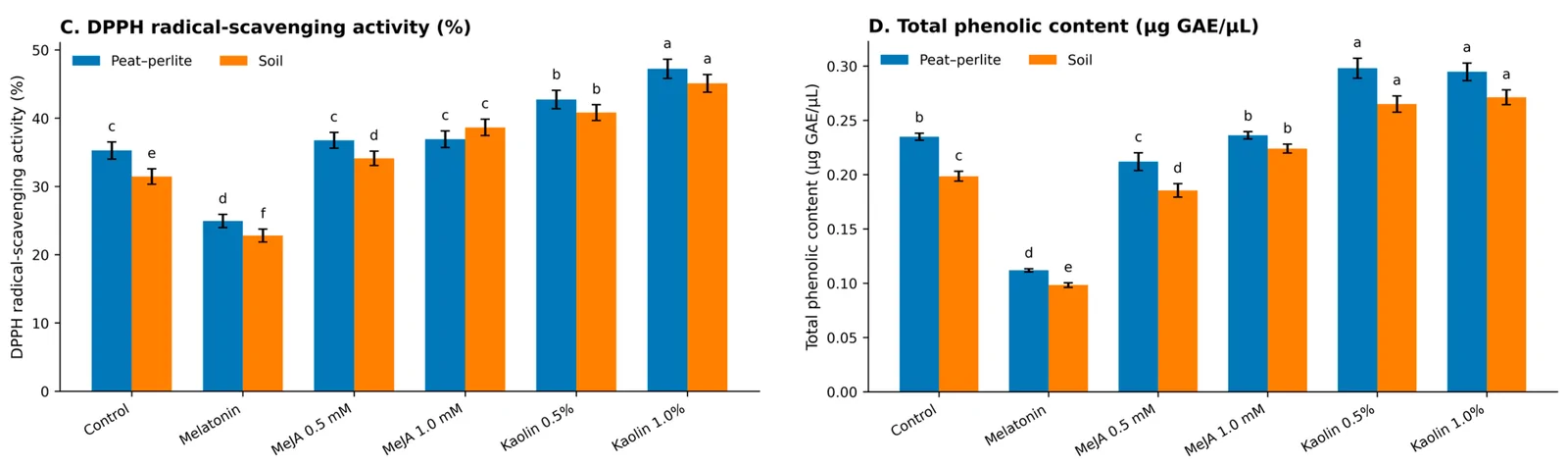
Comparative growth, physiological, and biochemical responses of lettuce to individually applied foliar treatments under the progressive high-temperature regime in peat–perlite and soil growing media. (**A**) Fresh plant weight; (**B**) relative water content (RWC); (**C**) DPPH radical-scavenging capacity; and (**D**) total phenolic content (TPC). Values are presented as mean ± SE. Different lowercase letters indicate significant differences among treatments within each growing medium according to the LSD test (*p* < 0.05).

**Table 1:** Morphological and growth parameters of lettuce grown in peat substrate.

Treatment	Plant Weight (g)	Root Weight (g)	Root Length (cm)	Root Diameter (mm)	Plant Height (cm)	Plant Dry Weight (g)	Root Dry Weight (g)
Kaolin 0.5%	11.15 ± 0.37 a	1.53 ± 0.14 a	7.41 ± 0.73 ab	7.26 ± 0.55 ab	16.36 ± 0.67 a	1.60 ± 0.31 a	0.13 ± 0.01 b
Kaolin 1.0%	11.61 ± 0.37 a	1.39 ± 0.19 ab	7.91 ± 0.31 ab	8.46 ± 0.75 a	16.79 ± 1.25 a	0.91 ± 0.09 b	0.07 ± 0.01 b
MeJA 0.5 mM	3.48 ± 0.37 bc	0.65 ± 0.17 b	5.31 ± 0.42 bc	4.06 ± 0.59 c	12.14 ± 0.63 bc	1.22 ± 0.15 ab	0.14 ± 0.03 b
MeJA 1.0 mM	9.86 ± 1.97 ab	1.09 ± 0.21 ab	8.21 ± 0.77 a	4.32 ± 0.79 bc	14.84 ± 1.07 ab	0.87 ± 0.08 b	0.55 ± 0.02 a
Melatonin	9.36 ± 0.85 ab	1.11 ± 0.11 ab	7.71 ± 0.68 ab	8.10 ± 0.81 a	13.79 ± 0.51 abc	0.79 ± 0.05 b	0.52 ± 0.01 a
Peat Control	1.13 ± 0.49 c	1.57 ± 0.18 a	4.74 ± 0.26 c	4.51 ± 0.40 bc	10.23 ± 0.73 c	1.69 ± 0.42 a	0.12 ± 0.03 b
CV (%)	8.45	7.12	5.85	6.30	4.92	5.10	6.50
LSD	1.14	0.15	0.72	0.69	1.23	0.65	0.03
*p*-value	<0.0001	0.0137	0.0009	<0.0001	0.0001	0.0255	<0.0001

Note: Different letters in the same column indicate statistically significant differences (LSD test, *p* < 0.05).

**Table 2:** Morphological and growth parameters of lettuce grown in soil substrate.

Treatment	Plant Weight (g)	Root Weight (g)	Root Length (cm)	Root Diameter (mm)	Plant Height (cm)	Plant Dry Weight (g)	Root Dry Weight (g)
Kaolin 0.5%	9.30 ± 2.94 ab	3.02 ± 0.42	8.40 ± 0.37	8.93 ± 0.60	17.90 ± 0.86 ab	1.28 ± 0.13 bc	0.63 ± 0.03 a
Kaolin 1.0%	7.30 ± 2.31 b	2.02 ± 0.27	9.55 ± 0.98	8.40 ± 1.09	15.75 ± 0.65 ab	2.37 ± 0.31 a	0.62 ± 0.06 a
Soil Control	8.69 ± 2.75 ab	2.38 ± 0.20	8.88 ± 0.33	8.81 ± 0.89	19.49 ± 0.88 a	1.41 ± 0.08 b	0.62 ± 0.03 a
MeJA 0.5 mM	6.66 ± 2.11 ab	2.65 ± 0.35	9.97 ± 1.01	9.17 ± 0.68	14.47 ± 1.46 b	0.50 ± 0.13 c	0.64 ± 0.03 a
MeJA 1.0 mM	12.34 ± 3.90 ab	2.37 ± 0.34	9.83 ± 0.66	9.47 ± 1.08	17.70 ± 0.66 ab	1.25 ± 0.18 bc	0.64 ± 0.06 a
Melatonin	18.14 ± 5.73 a	1.81 ± 0.19	9.88 ± 0.65	9.94 ± 1.11	18.95 ± 0.95 a	1.00 ± 0.13 bc	0.28 ± 0.06 b
CV (%)	9.15	8.20	6.10	5.90	5.25	7.40	6.80
LSD	1.69	0.35	1.02	0.96	1.62	0.17	0.07
*p*-value	0.0215	0.1599	0.6562	0.9214	0.0106	<0.0001	<0.0001

Note: Different letters in the same column indicate statistically significant differences (LSD test, *p* < 0.05).

**Table 3:** Physiological, biochemical, and color parameters of lettuce grown in peat substrate.

Treatment	Visual Scale	Leaf Temp (°C)	Membrane Leakage (%)	RWC (%)	TSS (°Brix)	pH	L*	a*	b*
Kaolin 0.5%	0.43 ± 0.18 b	21.49 ± 1.11	54.10 ± 12.37	56.06 ± 5.11 ab	1.75 ± 0.05	5.28 ± 0.02	19.71 ± 3.49 b	−6.54 ± 5.30	6.36 ± 7.14
Kaolin 1.0%	0.00 ± 0.06 b	21.06 ± 1.42	69.84 ± 4.09	40.78 ± 5.01 b	1.30 ± 0.10	5.31 ± 0.05	29.62 ± 4.22 ab	−17.27 ± 3.19	13.76 ± 9.43
MeJA 0.5 mM	0.71 ± 0.25 b	20.83 ± 0.87	56.94 ± 2.50	68.24 ± 9.90 a	1.58 ± 0.42	5.20 ± 0.02	24.02 ± 3.01 ab	−11.06 ± 2.13	31.17 ± 4.64
MeJA 1.0 mM	0.57 ± 0.30 b	20.90 ± 1.27	60.37 ± 1.63	72.40 ± 5.69 a	0.95 ± 0.05	5.38 ± 0.01	27.70 ± 2.30 ab	−6.42 ± 1.88	14.27 ± 2.27
Melatonin	0.00 ± 0.06 b	18.91 ± 0.54	74.56 ± 2.71	66.13 ± 6.26 a	1.70 ± 0.10	5.29 ± 0.03	37.26 ± 2.85 a	−11.79 ± 3.78	17.13 ± 1.89
Peat Control	2.71 ± 0.18 a	23.03 ± 0.56	75.66 ± 4.39	66.70 ± 2.37 a	1.50 ± 0.07	5.28 ± 0.04	23.10 ± 0.71 b	−6.64 ± 1.58	19.39 ± 2.44
CV (%)	9.50	4.20	6.80	5.90	7.10	0.80	6.40	7.80	8.20
LSD	0.12	1.57	7.89	6.48	0.18	0.08	3.06	1.38	2.48
*p*-value	<0.0001	0.2516	0.1445	0.0111	0.1395	0.0607	0.0116	0.2268	0.1211

Note: Different letters in the same column indicate statistically significant differences among treatments according to the LSD test (*p* < 0.05).

**Table 4:** Physiological, biochemical, and color parameters of lettuce grown in soil substrate.

Treatment	Visual Scale	Leaf Temp (°C)	Membrane Leakage (%)	RWC (%)	TSS (°Brix)	pH	L*	a*	b*
Kaolin 0.5%	0.40 ± 0.15	20.15 ± 0.72	45.01 ± 5.04	45.09 ± 4.77 b	1.15 ± 0.05	6.76 ± 0.06 ab	23.51 ± 2.02	−7.07 ± 1.79	20.25 ± 2.03 b
Kaolin 1.0%	0.70 ± 0.22	21.02 ± 1.10	42.88 ± 0.66	73.07 ± 6.53 a	1.40 ± 0.10	7.04 ± 0.00 a	23.50 ± 2.74	−12.86 ± 3.47	16.53 ± 3.63 b
Soil Control	0.60 ± 0.22	19.12 ± 0.33	53.37 ± 1.75	45.71 ± 7.42 b	1.55 ± 0.15	5.34 ± 0.02 c	28.92 ± 2.55	−11.75 ± 2.59	32.24 ± 3.89 a
MeJA 0.5 mM	0.30 ± 0.13	19.09 ± 0.36	43.71 ± 3.03	69.32 ± 6.14 a	1.40 ± 0.10	5.64 ± 0.17 c	24.38 ± 2.58	−12.26 ± 2.62	31.32 ± 3.64 a
MeJA 1.0 mM	0.20 ± 0.13	18.90 ± 0.47	37.42 ± 3.06	72.98 ± 4.99 a	1.50 ± 0.30	6.35 ± 0.27 b	21.42 ± 3.70	−9.87 ± 2.53	31.90 ± 5.18 a
Melatonin	0.30 ± 0.14	19.30 ± 0.31	39.05 ± 3.30	40.50 ± 9.93 b	1.05 ± 0.05	5.55 ± 0.05 c	16.33 ± 1.35	−8.88 ± 1.00	24.54 ± 2.80 ab
CV (%)	8.80	4.50	6.20	5.70	7.50	2.10	6.90	7.20	44.30
LSD	0.07	1.57	4.81	5.86	0.18	0.23	2.82	1.34	10.38
*p*-value	0.5420	0.8105	0.0931	0.0014	0.2415	0.0004	0.6210	0.6120	0.0098

Note: Different letters in the same column indicate statistically significant differences among treatments according to the LSD test (*p* < 0.05).

**Table 5:** DPPH and ABTS radical-scavenging capacity and total phenolic content of lettuce in peat–perlite substrate.

Treatment	DPPH (% Inhibition)	ABTS (% Inhibition)	Total Phenolic Content (µg GAE/µL)
Control	35.26 ± 1.25 c	95.03 ± 1.85 b	0.2349 ± 0.0032 b
Melatonin	24.93 ± 0.98 d	53.45 ± 1.50 d	0.1118 ± 0.0015 d
MeJA 0.5 mM	36.75 ± 1.15 c	79.93 ± 2.10 c	0.2119 ± 0.0082 c
MeJA 1.0 mM	36.92 ± 1.22 c	82.42 ± 1.90 c	0.2362 ± 0.0034 b
Kaolin 0.5%	42.74 ± 1.35 b	97.44 ± 1.45 a	0.2980 ± 0.0092 a
Kaolin 1.0%	47.24 ± 1.40 a	98.63 ± 1.30 a	0.2947 ± 0.0081 a
LSD (0.05)	2.19	3.04	0.0113
CV (%)	3.30	2.02	2.74
*p*-value	<0.001	<0.001	<0.001

Note: Different letters in the same column indicate statistically significant differences among treatments according to the LSD test (*p* < 0.05).

**Table 6:** DPPH and ABTS radical-scavenging capacity and total phenolic content of lettuce in soil substrate.

Treatment	DPPH (% Inhibition)	ABTS (% Inhibition)	Total Phenolic Content (µg GAE/µL)
Control	31.45 ± 1.12 e	88.20 ± 2.10 b	0.1985 ± 0.0045 c
Melatonin	22.80 ± 0.95 f	49.15 ± 1.85 e	0.0982 ± 0.0021 e
MeJA 0.5 mM	34.12 ± 1.05 d	76.50 ± 2.20 d	0.1854 ± 0.0062 d
MeJA 1.0 mM	38.65 ± 1.20 c	84.30 ± 1.95 c	0.2240 ± 0.0041 b
Kaolin 0.5%	40.82 ± 1.15 b	93.60 ± 1.50 a	0.2650 ± 0.0075 a
Kaolin 1.0%	45.10 ± 1.30 a	96.80 ± 1.40 a	0.2712 ± 0.0068 a
LSD (0.05)	2.02	3.30	0.0098
CV (%)	3.19	2.28	2.66
*p*-value	<0.001	<0.001	<0.001

Note: Different letters in the same column indicate statistically significant differences among treatments according to the LSD test (*p* < 0.05).

## Data Availability

The data presented in this study are available on request from the corresponding author.

## References

[1] Calvin K., Dasgupta D., Krinner G., Mukherji A., Thorne P.W., Trisos C., Romero J. (2023). IPCC, 2023: Climate Change 2023: Synthesis Report.

[2] Abouelsaad I., Lamlom S.F., El-Serafy R., Aboukila E., Alharbi A. (2026). Applied Heat-Stress Mitigation Strategies in Vegetable Crops: Toward Integrated Field-Scale Approaches. Horticulturae.

[3] Fahad S., Bajwa A.A., Nazir U., Anjum S.A., Farooq A., Zohaib A., Huang J. (2017). Crop production under drought and heat stress: Plant responses and management options. Front. Plant Sci..

[4] Lafta A., Turini T., Sandoya G.V., Mou B. (2017). Field evaluation of green and red leaf lettuce genotypes in California for heat tolerance. HortScience.

[5] Yang T., He S.P., Huo G.T., Tian M.L., Du L., Yang X., Wei S.W. (2025). LsNAC46 regulates heat-resistance and growth in lettuce (*Lactuca sativa* L.). Front. Plant Sci..

[6] Mittler R. (2017). ROS are good. Trends Plant Sci..

[7] Hasanuzzaman M., Bhuyan M.B., Zulfiqar F., Raza A., Mohsin S.M., Mahmud J.A., Fotopoulos V. (2020). Reactive oxygen species and antioxidant defense in plants under abiotic stress. Antioxidants.

[8] Hayat S., Hayat Q., Alyemeni M.N., Wani A.S., Pichtel J., Ahmad A. (2012). Role of proline under changing environments: A review. Plant Signal. Behav..

[9] Van Oosten M.J., Pepe O., De Pascale S., Silletti S., Maggio A. (2017). The role of biostimulants and bioeffectors as alleviators of abiotic stress in crop plants. Chem. Biol. Technol. Agric..

[10] Schindler U., Lischeid G., Müller L. (2017). Hydraulic Performance of Horticultural Substrates-3. Impact of Substrate Composition and Ingredients. Horticulturae.

[11] Arnao M.B., Hernández-Ruiz J. (2019). Melatonin: A new plant hormone and/or a plant master regulator?. Trends Plant Sci..

[12] Jahan M.S., Guo S., Sun J., Shu S., Wang Y., Abou El-Yazied A., Hasan M.M. (2021). Melatonin-mediated photosynthetic performance of tomato seedlings under high-temperature stress. Plant Physiol. Biochem..

[13] Wasternack C., Song S. (2017). Jasmonates: Biosynthesis, metabolism, and signaling by proteins activating and repressing transcription. J. Exp. Bot..

[14] Ali M.S., Baek K.H. (2020). Jasmonic acid signaling pathway in response to abiotic stresses in plants. Int. J. Mol. Sci..

[15] Kim H.J., Fonseca J.M., Choi J.H., Kubota C. (2007). Effect of methyl jasmonate on phenolic compounds and carotenoids of romaine lettuce (*Lactuca sativa* L.). J. Agric. Food Chem..

[16] Glenn D.M. (2012). The mechanisms of plant stress mitigation by kaolin-based particle films and applications in horticultural and agricultural crops. HortScience.

[17] Chen L., Xu M., Liu C., Hao J., Fan S., Han Y. (2022). LsMYB15 regulates bolting in leaf lettuce (*Lactuca sativa* L.) under high-temperature stress. Front. Plant Sci..

[18] Zhao X., Sui X., Zhao L., Gao X., Wang J., Wen X., Li Y. (2022). Morphological and physiological response mechanism of lettuce (*Lactuca sativa* L.) to consecutive heat stress. Sci. Hortic..

[19] Lee D.Y., Park D.Y., Park H.C. (2024). Exogenous methyl jasmonate mediates tolerance of heat stress in Korean fir (*Abies koreana*). Plant Biotechnol. Rep..

[20] Sun C., Meng S., Wang B., Zhao S., Liu Y., Qi M., Wang Z., Yin Z., Li T. (2023). Exogenous melatonin enhances tomato heat resistance by regulating photosynthetic electron flux and maintaining ROS homeostasis. Plant Physiol. Biochem..

[21] Terán F., Vives-Peris V., López-Climent M.F., Gómez-Cadenas A., Pérez-Clemente R.M. (2024). Palliative Effects of Kaolin on Citrus Plants Under Controlled Stress Conditions of High Temperature and High Light Intensity. J. Plant Growth Regul..

[22] Wu J., Wang Y., Zhang L., Liu X. (2024). Mechanisms of jasmonic acid and methyl jasmonate in regulating stress responses and quality of vegetables: A review. Plants.

[23] Liu X., Ardo S., Bunning M., Parry J., Zhou K., Stushnoff C., Stoniker F., Yu L., Kendall P. (2007). Total phenolic content and DPPH radical scavenging activity of lettuce (*Lactuca sativa* L.) grown in Colorado. LWT—Food Sci. Technol..

[24] Wei S., Zhang L., Huo G., Ge G., Luo L., Yang Q., Long P. (2021). Comparative transcriptomics and metabolomics analyses provide insights into thermal resistance in lettuce (*Lactuca sativa* L.). Sci. Hortic..

[25] Hoagland D.R., Arnon D.I. (1950). The water-culture method for growing plants without soil. Calif. Agric. Exp. Stn. Circ..

[26] Brand-Williams W., Cuvelier M.E., Berset C. (1995). Use of a free radical method to evaluate antioxidant activity. LWT-Food Sci. Technol..

[27] Re R., Pellegrini N., Proteggente A., Pannala A., Yang M., Rice-Evans C. (1999). Antioxidant activity applying an improved ABTS radical cation decolorization assay. Free Radic. Biol. Med..

[28] Singleton V.L., Rossi J.A. (1965). Colorimetry of total phenolics with phosphomolybdic-phosphotungstic acid reagents. Am. J. Enol. Vitic..

[29] Slinkard K., Singleton V.L. (1977). Total phenol analysis: Automation and comparison with manual methods. Am. J. Enol. Vitic..

